# Identification of Novel Cetacean Poxviruses in Cetaceans Stranded in South West England

**DOI:** 10.1371/journal.pone.0124315

**Published:** 2015-06-05

**Authors:** James Barnett, Akbar Dastjerdi, Nick Davison, Rob Deaville, David Everest, Julie Peake, Christopher Finnegan, Paul Jepson, Falko Steinbach

**Affiliations:** 1 Animal and Plant Health Agency (APHA), Polwhele, Truro, United Kingdom; 2 Virology Department, Science Division, APHA, Weybridge, Addlestone, United Kingdom; 3 UK Cetacean Strandings Investigation Programme, Institute of Zoology, London, United Kingdom; 4 Pathology Department, Science Division, APHA, Weybridge, Addlestone, United Kingdom; 5 Environment and Sustainability Institute, University of Exeter Cornwall Campus, Penryn, United Kingdom; 6 Scottish Marine Animal Stranding Scheme, SRUC Veterinary Services, Inverness, United Kingdom; 7 School of Veterinary Medicine, Faculty of Health and Medical Sciences, University of Surrey, Guildford, United Kingdom; University of Minnesota, UNITED STATES

## Abstract

Poxvirus infections in marine mammals have been mainly reported through their clinical lesions and electron microscopy (EM). Poxvirus particles in association with such lesions have been demonstrated by EM and were previously classified as two new viruses, cetacean poxvirus 1 (CePV-1) and cetacean poxvirus 2 (CePV-2). In this study, epidermal pox lesions in cetaceans stranded in South West England (Cornwall) between 2008 and 2012 were investigated by electron microscopy and molecular analysis. PCR and sequencing of a highly conserved region within the viral DNA polymerase gene ruled out both parapox- and orthopoxviruses. Moreover, phylogenetic analysis of the PCR product clustered the sequences with those previously described as cetacean poxviruses. However, taking the close genetic distance of this gene fragment across the family of poxviridae into account, it is reasonable to postulate further, novel cetacean poxvirus species. The nucleotide similarity within each cluster (tentative species) detected ranged from 98.6% to 100%, whilst the similarity between the clusters was no more than 95%. The detection of several species of poxvirus in different cetacean species confirms the likelihood of a heterogeneous cetacean poxvirus genus, comparable to the heterogeneity observed in other poxvirus genera.

## Introduction

Poxvirus infections in marine mammals have mainly been reported through their clinical lesions and EM. In pinnipeds, where infections are associated with cutaneous and occasionally oral nodule formation, the type of poxvirus detected almost invariably had the morphological characteristics of parapoxviruses [1, 2, 3, 4]. A parapoxvirus has been isolated from pox-like lesions in grey seals (*Halichoerus grypus*) [5] and has been demonstrated by polymerase chain reaction (PCR) in harbour seals (*Phoca vitulina*) [6].

In cetaceans, the cutaneous manifestations of reported poxvirus infections are hyperpigmented skin lesions, variously described as ‘ring’, ‘pinhole’ and ‘tattoo’ lesions. Poxvirus particles in association with such lesions have been demonstrated by EM [7, 8, 9, 10] and initially characterised via PCR and sequencing by Bracht *et al*. [11], who suggested two new viruses, cetacean poxvirus 1 (CePV-1) and cetacean poxvirus 2 (CePV-2). That study included 92 cetaceans of various species, mainly sampled in stranding programmes in Florida and Alaska between 2000 and 2004. Molecular characterisation was primarily based on a PCR amplification of the viral DNA polymerase gene (homologues of VVE9L, LSDV039, SPV036) resulting in a 546kb fragment in 10 samples from five cetacean (four dolphin and one whale) species [11, 12].

Skin lesions consistent with poxvirus infections, have been reported in cetaceans stranded in UK waters, including harbour porpoises (*Phocoena phocoena*), striped dolphins (*Stenella coeruleoalba*), a white-beaked dolphin (*Lagenorhynchus albirostris*) and a long finned pilot whale (*Globicephala melas*) [13, 14]. Most recently, a retrospective study using archived material (2000–2008) from UK stranded cetaceans demonstrated CePV-1 viruses using PCR as described above [15]. In the current study, pox-like lesions of cetaceans stranded in south west England (Cornwall) between 2008 and 2012 were investigated by EM, histopathology, and PCR followed by sequencing.

## Material and Methods

Work was carried out under The Conservation of Habitats and Species Regulations using a class license (WML-CL01) for the possession and transport of dead specimens of wild plant and animal species issued to the investigators. No approval was necessary under animal research since all animals were dead prior to study and not part of any regulated procedure. Stranded cetaceans considered in suitable condition for post mortem examination were retrieved for detailed post mortem examination between August 2008 and September 2012 (S1 Table) using standard protocols [16]. Post-mortem investigations were carried out under the UK *Cetacean Strandings Investigation Programme*. Skin lesions and other tissue sections were stored in 10% buffered formalin for histopathological investigations. Fresh samples from suspected poxvirus lesions were examined further with negative contrast stain Transmission Electron Microscopy (TEM) in order to detect pox-like viral particles.

### TEM analyses

Support grids of 3mm diameter Copper/Rhodium (100μ mesh) were used for the analyses. Each grid was pre-treated by immersion into 0.4% (w/v) formvar in chloroform, followed by carbon coating, to provide a stable sample platform. Treatment was then completed, by subjecting each grid to plasma glow discharge, to ensure the grids were highly hydrophilic. Each sample (approx. 0.50g) was ground in 2cm^3^ of 0.1M Sorenson’s phosphate buffer (pH 6.6), to form a suspension. An aliquot of each sample suspension (50μl) was pipetted onto a piece of dental wax and a support grid was then placed copper side upwards onto the aliquot for 30 seconds and excess sample removed by wicking dry. Each grid was then placed as before onto a drop of 2% phosphotungstic acid (pH 6.6) for 10 seconds to counter-stain the grid and again excess stain removed by wicking dry. Analysis of each sample was undertaken on a Phillips CM10, or CM100 transmission electron microscope at x34,000 magnification at 80 kilovolts. Confirmation of viral particle presence was by size, shape and available surface morphology at x92,000 magnification and each positive sample was recorded on a set of digital images taken on a Deben 1K camera. Analysis time was standardised as taking 20 minutes viewing of the sample grid or checking 25 grid squares whichever was shortest, to observe if any virus particles were present. For comparison, samples from the collection of ortho- and parapoxviruses held by the AHVLA as part of their Diseases of Wildlife Scheme (*AHVLA DoWS)*, now managed as part of the GB Diseases of Wildlife Surveillance Partnership were included in the study.

### Histopathology

In all but one (M120-02-12, S1 Table) case where poxviruses were confirmed by TEM, samples from lesions were examined histologically. For this purpose, 4–6 μm thin sections were stained with haematoxylin and eosin (H&E).

### PCR and sequencing

Frozen sub-samples of lesions, positive for pox-like viral particles lesions by TEM, were subjected to PCR analyses, along with a small number of suspect TEM negative pox lesions. The PCR primers used in this study target a highly conserved region within the DNA polymerase gene of poxviruses [12]. The primers, DNA-pol FP 5’ ATA CAG AGC TAG TAC ITT AAT AAA AG 3’ and DNA-pol RP 5’ CTA TTT TTA AAT CCC ATT AAA CC 3’, amplify a 543 nucleotide segment of the polymerase gene. The PCR was performed using the QIAGEN Fast Cycling PCR kit at 95°C for 5 minutes and 40 cycles of 95°C for 30 seconds, 50°C for 30 seconds and 72°C for 1 minute followed by an elongation step of 72°C for 7 minutes. The amplified products were run on a 1.5% standard agarose gel and the amplicons of expected size were cut and cleaned using MiniElute gel extraction kit (Qiagen). Direct sequencing reactions were performed using BigDye Terminator v3.1 Cycle Sequencing kit (Life Technologies) as per manufacturer’s instructions and the labelled products were sequenced on an ABI 3130xL genetic analyser platform (Life Technologies). For investigating the presence of cetacean parapoxviruses the primers PPP-1, PPP-3 and PPP-4 were used as described [17].

For phylogenetic analysis, representative virus strains from each genus in the sub-family *Chordopoxvirinae* were compiled from GenBank and included in the analysis. Sequences were aligned using the MegAlign software (ClustalW) implemented in the DNASTAR LaserGene 9.0 package. The alignments were imported into MEGA 5.03 software for phylogenetic tree construction using the neighbour-joining method. The confidence values in tree grouping were calculated using bootstrapping (2000 replicates).

## Results and Discussion

The current study describes the detection and characterisation of poxviruses and lesions induced in cetaceans stranded on the coast of Cornwall, south west England between 2008 and 2012. A significant correlation of poxvirus infection with length (age) has been reported in some species, with neonates and juveniles not exhibiting poxvirus lesions until a certain body length (species dependent), which may be at least partly explained by maternal antibodies conveying protection from infection [10]. Conversely, lesions are then observed in (post)-weaning individuals correlated with an increased interaction of juveniles with other potentially infected cetaceans. In this study, the lengths of the eight poxvirus-positive animals were in excess of weaning lengths (S1 Table) for all three species [18, 19], i.e. consistent with this hypothesis.

**Fig 1:**
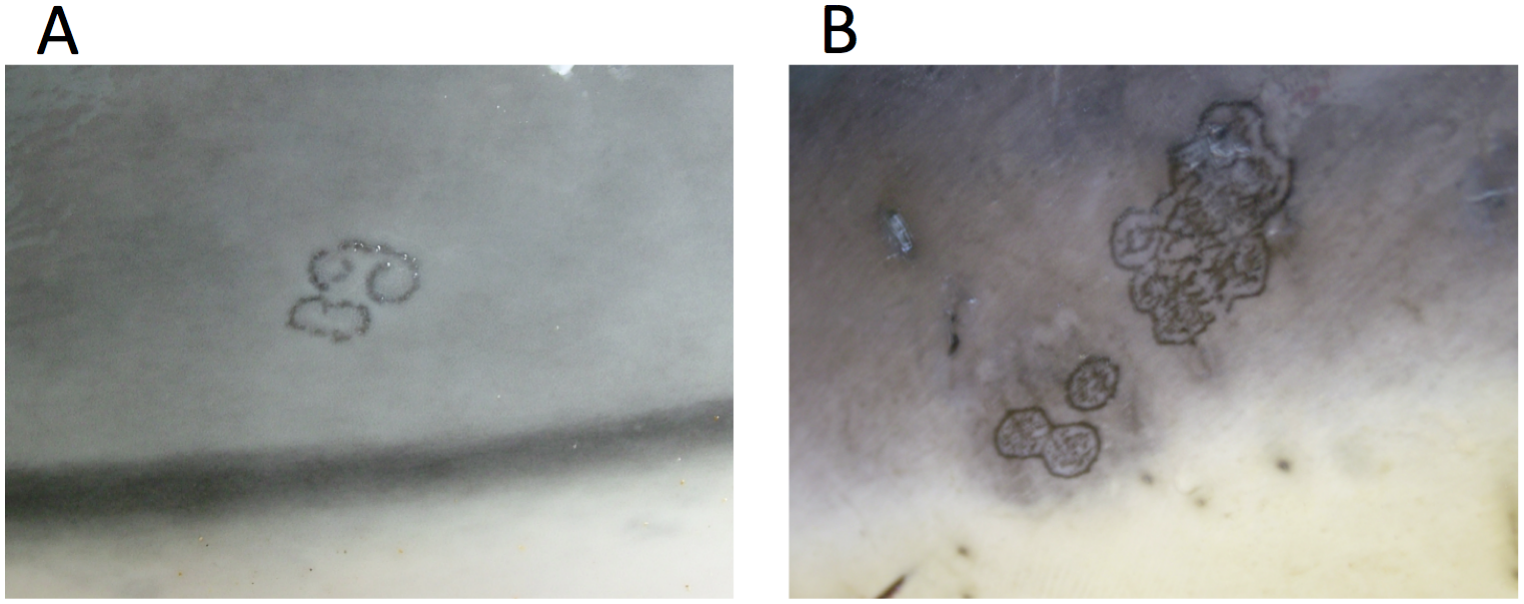
Tattoo lesions seen in cetacean poxvirus-infected animals. Tattoo lesion on the thorax of a juvenile male striped dolphin (*Stenella coeruleoalba*) (a) and tattoo lesion on the thorax of a juvenile female common dolphin (*Delphinus delphis*) (b).

**Fig 2:**
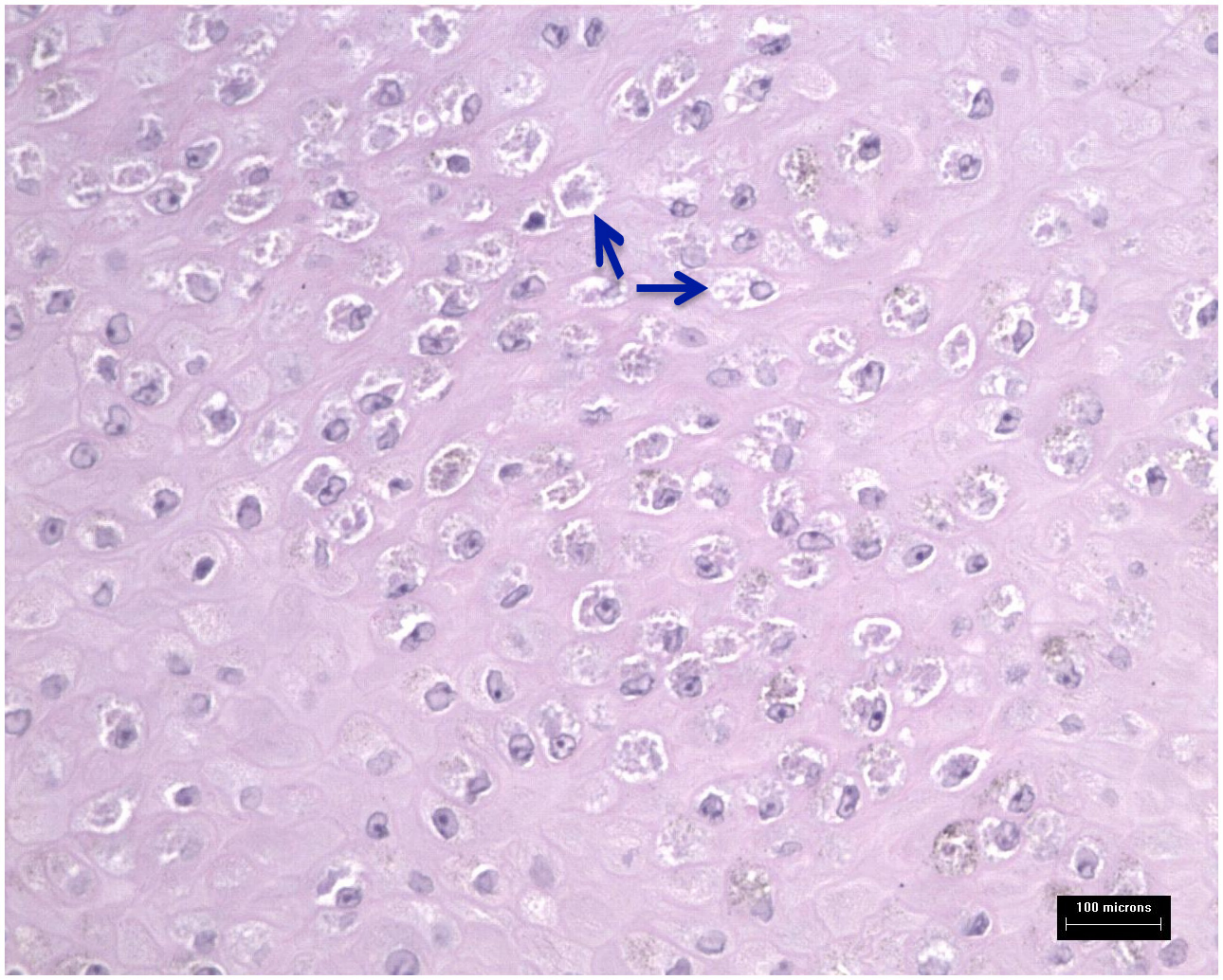
Haematoxylin and eosin staining of skin sections positive for cetacean poxviruses by TEM and PCR. Focal areas of cytoplasmic vacuolation are within the stratum intermedium of the skin (arrows).

Poxvirus particles were detected in typical tattoo-like skin lesions (Fig 1) by EM in samples from eight stranded cetaceans of three species, consisting of four short-beaked common dolphins (*Delphinus delphis*), three harbour porpoises (*Phocoena phocoena*) and one striped dolphin (*Stenella coeruleoalba*). Seven of these cases were further examined histopathologically, where a microscopic appearance characteristic of poxviral skin disease was observed in all cases (S1 Table). Overall, focal areas of cytoplasmic vacuolation were detected within the stratum intermedium of the skin. Less clearly visible were small round, pale eosinophilic, intra-cytoplasmic inclusion bodies (Fig 2).

**Fig 3:**
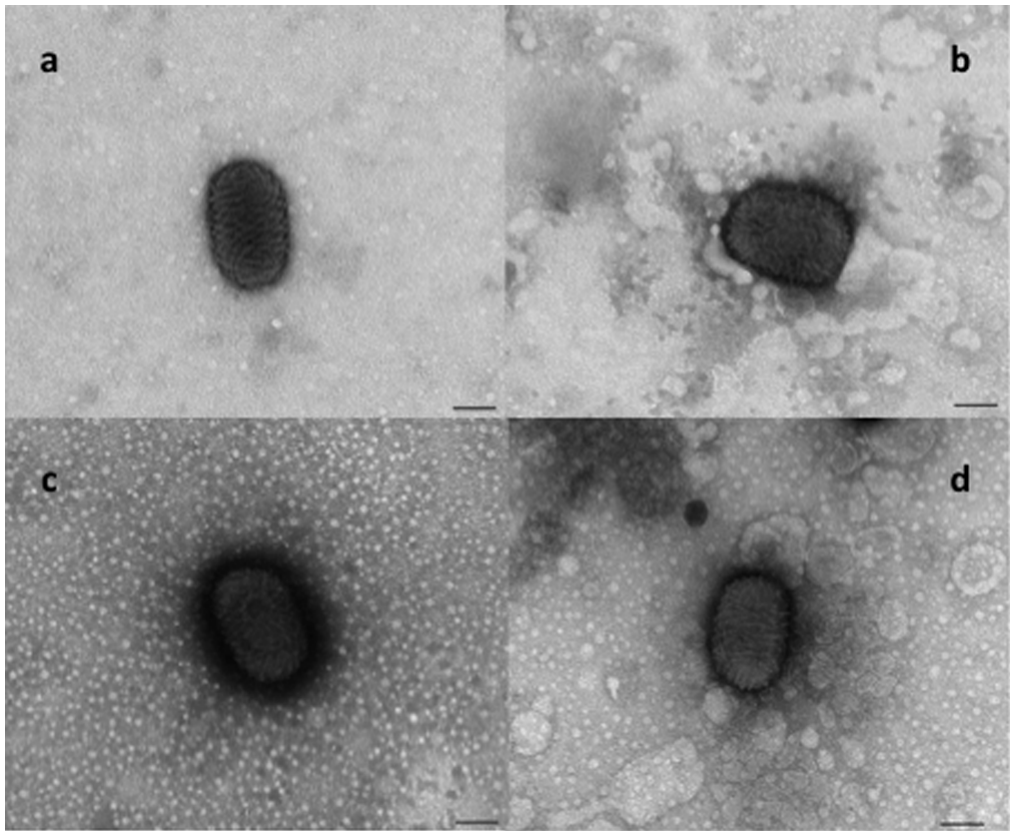
Electron microscopic analysis of cetacean poxviruses. Viral particles from tattoo lesions of a juvenile male striped common dolphin (c) and a juvenile female harbour porpoise (d). By their size and ovoid shape they resemble parapoxviruses, such as the sheep parapoxvirus displayed for comparison (a). Their surface morphology, however, more resembles a ball of string, typical of orthopoxviruses (b). (Original magnification 92,000x; *Bar = 100nm*)

The poxvirus particles observed in EM, initially by size and ovoid shape, were more consistent with the phenotype of parapoxviruses. At higher magnifications however, their surface morphology was characteristic of the disorderly structure of an orthopoxvirus (Fig 3). The combination of both shapes may have misled earlier investigations before the molecular age resulting in the assumption of orthopoxvirus infections in cetaceans [9].

**Fig 4:**
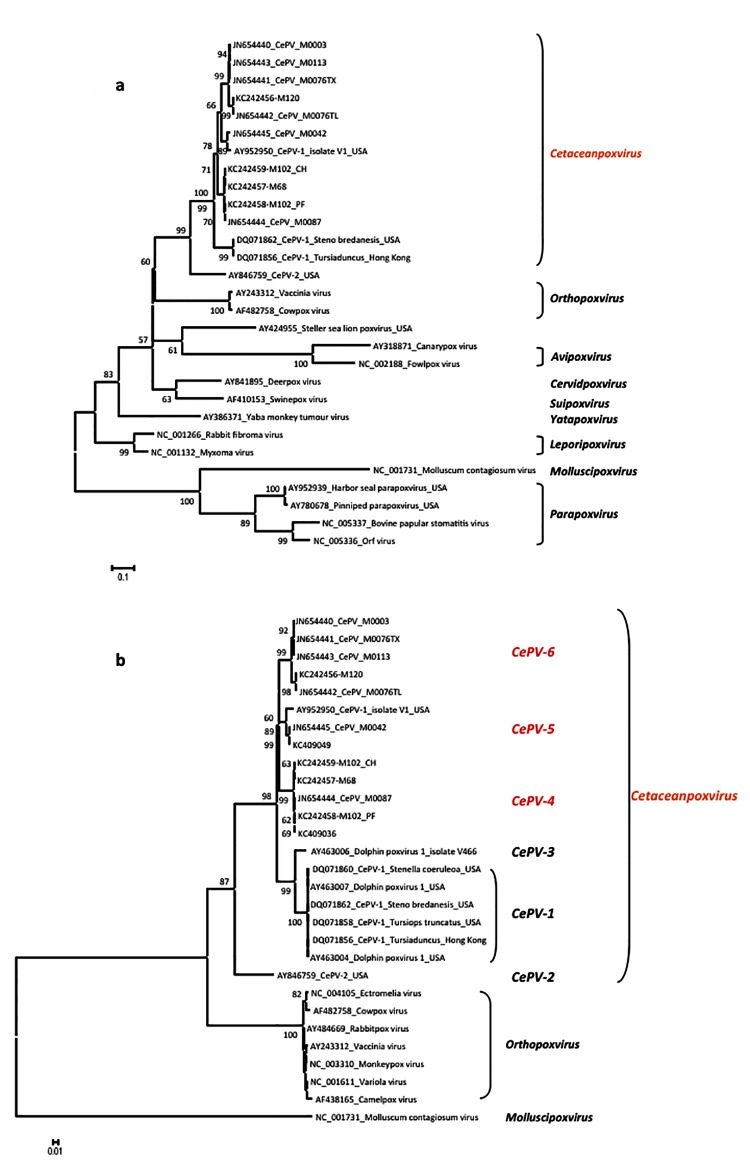
Phylogenetic analysis of the polymerase gene of cetacean poxviruses and reference sequences available from GenBank representing the known genera in the sub-family Chordopoxvirinae. The phylogenetic tree was constructed by MEGA 5 software and the confidence levels were calculated using bootstrapping (2000 replicates). Only bootstrap values greater than 50 are shown. As seen in the overview (Fig 4a), cetacean poxviruses do not cluster with any other known genus, substantiating the notion that they are to be regarded as a separate one. While parts of the overall topology obtain a relatively low statistical support (after extensive bootstrapping) it resembles the established classification of poxvirus genera and the differences in sequences (both in % and reflected in branch length) support this notion. In more detail (Fig 4b) and taking cetacean poxviruses from other published studies [11,15] into account, six separate species/clusters of poxviruses are recognisable.

Material from all eight animals where EM had identified poxvirus particles was further analysed by PCR. For two of the animals, samples were analysed from two lesions on each animal. The presence of poxvirus-specific DNA was demonstrated by PCR in all cases. Sequencing revealed no evidence for parapoxvirus (as reported in pinnipeds) or orthopoxvirus (in terrestrial mammals). Instead, all sequences clustered in closer proximity to those previously described as cetacean poxvirus (CePV)-1 and -2 (Fig 4a) underpinning the notion that cetacean poxviruses form a separate genus.

Comparing the sequences in more detail, focussing on orthpoxviruses as a reference genus infecting a wide variety of mammals and Molluscum contagiosum virus as a mammalian poxvirus out-group, it is evident that the cetacean poxviruses detected here and in the previous study by Bracht *et al*. [11] fall into several clusters (Fig 4b). Within these, the CePV-2 previously detected in a bowhead whale (*Balaena mysticetus*) stands out singularly and six sequences from the US cluster described as CePV-1 appear identical to each other. One more sequence from a bottlenose dolphin (*Tursiops aduncus*) is significantly different to these and in line with our findings should be considered a separate cetacean poxvirus species (CePV-3). The sequences described here and most recently by Blacklaws *et al*. [15], as well as two of the previous sequences described by Bracht *et al*. [11] form further clusters, which are designated as CePV- 4 to 6.

Taking the close genetic relationship of the polymerase gene within other genera such as orthopox or capripox into account, it is reasonable to postulate that these clusters represent cetacean poxvirus species (CePV-1 to -6). The nucleotide identity within the genus orthopoxvirus for example ranges from 97.5–99.5%. In comparison, the nucleotide identity within the CePV clusters 4–6 is 98–100% and between the clusters 3–5 is 93.9–95.3%. The identity between CePV clusters 4–6 and CePV-1 is 89.1–91.6% and between CePV-2 and all others is 81.6–84.1%. In summary, the detection of several distinct sequences of poxvirus in the eight individuals of three cetacean species confirms that the cetacean poxvirus genus has comparable heterogeneity to the orthopoxvirus genus.

In this context, the results in the two cases where two samples were obtained from an individual animal are also noteworthy. In animal M102-09-11 (a harbour porpoise, *Phocoena phocoena*) the samples are most likely to be the same poxvirus species, falling into the tight clustering of CePV-5 (with 99.7% identity). In animal M76-11-09 however (a short-beaked common dolphin, *Delphinus delphis*), the two sequences (JN654441 from the thoracic region and JN654442 from the tail) are only 98% identical and fall into two different subsets within the CePV cluster 4. In this instance, it seems possible that animal M76-11-09 had actually been infected by two different cetacean poxvirus strains; as such a strong genetic drift seems unlikely within an infected individual.

Given that the number of marine viruses has previously been estimated at around ~4x10^30^ viruses (including all plant viruses and phages [20], the social interactions and long range that some species of cetacean travel, it is perhaps not surprising that cetaceans can be exposed to multiple viral infections. In the absence of the recognition of the cetacean poxvirus genus and the absence of a species definition, it is presently impossible to delineate the true number of CePV species, which will also require further characterisation of the viruses.

The species specificity of poxviruses is variable. Some genera seem to be highly restricted to individual hosts (swinepox), whereas others, such as orthopoxviruses infect a range of species. The cetacean poxviruses described here and elsewhere [11, 15] seem to be highly restricted to cetaceans, but thus far no clear pattern regarding species-specificity can be delineated. Considering the potentially wide spatial separation of cetacean populations and, in some species, individuals, it would be beneficial for the viruses to sustain a less restricted specificity [21].

In some reviews and studies, cetacean poxvirus infection is reported as generally not causing significant clinical disease in infected animals [7, 9, 22]. While van Bressem and van Waerebeek [10] reported the occasional association of poxvirus lesions with wounds or scars, this was not evident in this survey, although wounds are known to heal particularly quickly in cetaceans due to the extremely high turnover of dolphin epidermal cells [8]. It has been suggested elsewhere that poxvirus infections in cetaceans may be associated with stressful conditions such as poor water quality, environmental contamination and underlying ill health [7, 23]. Furthermore, acute and generalised, and also occasionally fatal infections have been reported in some instances [22, 24, 25]. Therefore, the pathogenesis of cetacean poxvirus infections is unclear and needs to be investigated further. To our knowledge, no studies have looked across different organ systems to detect poxvirus DNA in the absence of lesions. The absence of poxviruses in many investigated skin lesions also questions the role of cetacean poxviruses in their generation. Without further analyses (including the investigation of potentially related factors such as toxicology), it is difficult to comment on the role of environmental factors in the development of lesions in these animals. High contaminant levels have been found for example in stranded bottlenose dolphins (*Tursiops truncatus*) in south west England [26] and further studies are needed to investigate potential relationships between chemical or other environmental stressors and cetacean health, including disease related to poxvirus infection.

## Supporting Information

S1 Table Case details for animals with lesions confirmed for cetacean poxvirus. (DOC)
