# Supplementary material for: Identification of Novel Cetacean Poxviruses in Cetaceans Stranded in South West England
Source: PLoS One. 2015 Jun 5;10(6):e0124315. doi: 10.1371/journal.pone.0124315 (PMC4457422; doi:10.1371/journal.pone.0124315)
Supplement: S1 Table — (DOC) [file pone.0124315.s001.doc]

## Table S1: Case details for animals with lesions confirmed for cetacean poxvirus

| **Species** | **Month and year of sampling** | **Reference number** | **Age class, sex, body length** | **Size and distribution of tattoo lesions** | **Histopathological findings** | **Summary of other  post mortem findings** | **GenBank**  **Accession No** | **Cetacean poxvirus cluster** |
| --- | --- | --- | --- | --- | --- | --- | --- | --- |
| *Stenella coeruleoalba*  (Striped dolphin) | August 2008 | M42-08-08 | Juvenile male  1.93 m | 1-4 cm diameter tattoo and ring lesions on the right mandible, right axilla, right thorax and flank, leading edge of the dorsal fin and ventral midline near the genital slit. | Many cells in the inner epidermal layer with peripherally distributed/ crescentric. Some of these cells also had amphophilic/ eosinophilic bodies within the cytoplasm. The parakeratotic layer of the epidermis did not appear abnormal. | Poor nutritive state, mild parasitism, enteritis, interspecific rake marks, *Brucella ceti* isolated from cerebrospinal fluid. | JN654445 | CePV-5 |
| *Phocoena*  *phocoena*  (Harbour porpoise) | January 2009 | M87-01-09 | Juvenile female  1.26 m | 1cm diameter, single ring lesion on the right mandible. | Marked focal thickening of epidermal rete pegs associated with mild spongiosis in inner epidermis. Some epidermal cells had crescentric nuclei and intracytoplasmic amphophilic inclusion bodies were occasionally seen. Small clusters of (mainly mononuclear) inflammatory cells were present within dermal papillae. | Good nutritive state, lesions consistent with bycatch, parasitism. | JN654444 | CePV-4 |
| *Delphinus delphis*  (Short-beaked common dolphin) | *November* 2009 | M76-11-09 | Juvenile female  1.70m | Up to 1cm diameter multiple tattoo lesions, in some cases coalescing into larger lesions, on the right and left thorax and near the ventral midline of the tailstock. | Areas of increased vascular congestion within the dermal pegs and areas of hyperkeratosis and parakeratosis and areas of hyperpigmentation. There were focal areas (zones) of cytoplasmic vacuolation within the stratum intermedium associated with pale eosinophilic, small round, intra-cytoplasmic inclusion bodies. | Good nutritive state, lesions consistent with bycatch, gastritis, hepatopathy. | JN654441 & JN654442 | CePV-6 |

| *Delphinus delphis*  (Short-beaked common dolphin) | November 2010 | M3-11-10 | Subadult male  2.04m | One irregular tattoo lesion with an approximate diameter of 5cm, on the right thorax and two approximately 1cm diameter tattoo lesions on the right tail stock. | On some foci there were small and weakly eosinophilic intracytoplasmic inclusion bodies within the stratum intermedium. In other areas, there was cytoplasmic vacuolation of epidermal cells of the stratum intermedium with crescent-shaped peripherally-located nuclei. The cells of the stratum externum appeared increased compressing the cells downwards without overall elevation of the skin layer. Occasional breaks appeared in the stratum externum extending down into the stratum intermedium. These were often associated with eosinophilic cell debris and aggregates of basophilic material (bacteria?). Basophilic material (bacteria?) was also widely seen in dermal pegs but without associated cellular or vascular responses – these may be post-mortem invaders. | Moderate nutritive state, lesions consistent with bycatch. | JN654440 | CePV-6 |
| --- | --- | --- | --- | --- | --- | --- | --- | --- |
| *Delphinus delphis*  *(* Short-beaked common dolphin*)* | February 2011 | M113-02-11 | Adult male  2.03m | Three tattoo lesions on the left thorax/flank (3 x 3.5cm, 6 x 3.5cm and 1 x 1.5cm); two were irregular and the third was oval in shape. | There were regions of small and weakly eosinophilic intracytoplasmic inclusion bodies within the stratum intermedium associated with crescent-shaped peripherally-located nuclei. Occasional partial thickness (breaks) had appeared in the stratum externum and stratum intermedium along the section. In some foci the rete pegs appeared congested with some polymorphs visible within the congested blood vessels. | Good nutritive state. Live stranded, cause unclear on examination. Euthanased | JN654443 | CePV-6 |

| *Phocoena*  *phocoena*  (Harbour porpoise) | September 2011 | M102-09-11 | Juvenile male  1.40m | One near oval tattoo lesion on the left pectoral fin (1.6 x 1cm). One circular granulating wound (2cm diameter) with a central depression on the right aspect of the caudal head. | Occasional partial thickness (breaks) had appeared mainly in the stratum externum. In some foci the rete pegs appeared congested and mixed mononuclear/polymorphs were visible within the congested blood vessels. Crescent-shaped peripherally-located nuclei were common within the stratum intermedium in this section but eosinophilic intracytoplasmic inclusion bodies were present but rare. | Poor nutritive state. Numerous healed and partially healed skin wounds. Severe parasitism of the respiratory tract and peribullar spaces. *Brucella ceti* isolated from lung and liver. | KC242458 & KC242459 | CePV-4 |
| --- | --- | --- | --- | --- | --- | --- | --- | --- |
| *Delphinus delphis*  (Short-beaked common dolphin) | February 2012 | M120-02-12 | (Sub)adult female  1.86m | A number of slightly depressed, 1cm diameter ring lesions present to the left of the anus. | No histopathology undertaken. | Moderate to poor nutritive state. Asymmetrically congested lungs and fresh abrasions on the rostrum and one pectoral fin suggestive of live stranding, the cause of which was unclear on examination. | KC242456 | CePV-6 |
| *Phocoena phocoena*  (Harbour porpoise) | April 2012 | M68-04-12 | Juvenile male  1.35m | Three irregular dark pigmented areas, 1 to 2cm diameter, present over the right mandible and maxilla. | Areas of increased vascular congestion within the dermal pegs and areas of hyperkeratosis and parakeratosis. There were focal areas (zones) of cytoplasmic vacuolation within the stratum intermedium associated with hyperpigmentation and pale eosinophilic, small round, intra-cytoplasmic inclusion bodies. | Good nutritive state, lesions consistent with *Tursiops truncatus* attack, severe parasitism of the respiratory tract and associated pneumonia. | KC242457 | CePV-4 |
